# Nitrogen Nutrition Modulates the Response to *Alternaria brassicicola* Infection via Metabolic Modifications in *Arabidopsis* Seedlings

**DOI:** 10.3390/plants13040534

**Published:** 2024-02-15

**Authors:** Thibault Barrit, Elisabeth Planchet, Jérémy Lothier, Pascale Satour, Sophie Aligon, Guillaume Tcherkez, Anis M. Limami, Claire Campion, Béatrice Teulat

**Affiliations:** 1 Institut Agro, University of Angers, INRAE, IRHS, SFR QUASAV, 49000 Angers, France; thibault.barrit@gmail.com (T.B.); elisabeth.planchet@univ-angers.fr (E.P.); jeremy.lothier@univ-angers.fr (J.L.); pascale.satour@univ-angers.fr (P.S.); sophie.aligon@agrocampus-ouest.fr (S.A.); guillaume.tcherkez@anu.edu.au (G.T.); anis.limami@univ-angers.fr (A.M.L.); claire.campion@univ-angers.fr (C.C.); 2 Research School of Biology, Australian National University, Canberra, ACT 2601, Australia

**Keywords:** ammonium, necrotrophic fungus, nitrate, polyamines, seed germination, seedling development

## Abstract

Little is known about the effect of nitrogen nutrition on seedling susceptibility to seed-borne pathogens. We have previously shown that seedlings grown under high nitrate (5 mM) conditions are less susceptible than those grown under low nitrate (0.1 mM) and ammonium (5 mM) in the Arabidopsis-*Alternaria brassicicola* pathosystem. However, it is not known how seedling metabolism is modulated by nitrogen nutrition, nor what is its response to pathogen infection. Here, we addressed this question using the same pathosystem and nutritive conditions, examining germination kinetics, seedling development, but also shoot ion contents, metabolome, and selected gene expression. Nitrogen nutrition clearly altered the seedling metabolome. A similar metabolomic profile was observed in inoculated seedlings grown at high nitrate levels and in not inoculated-seedlings. High nitrate levels also led to specific gene expression patterns (e.g., polyamine metabolism), while other genes responded to inoculation regardless of nitrogen supply conditions. Furthermore, the metabolites best correlated with high disease symptoms were coumarate, tyrosine, hemicellulose sugars, and polyamines, and those associated with low symptoms were organic acids (tricarboxylic acid pathway, glycerate, shikimate), sugars derivatives and β-alanine. Overall, our results suggest that the beneficial effect of high nitrate nutrition on seedling susceptibility is likely due to nutritive and signaling mechanisms affecting developmental plant processes detrimental to the pathogen. In particular, it may be due to a constitutively high tryptophan metabolism, as well as down regulation of oxidative stress caused by polyamine catabolism.

## 1. Introduction

Nitrogen (N) availability is crucial for plant growth and, for example, nitrate (NO_3_^−^)-containing fertilizers have been widely used to this day to improve yields. However, excessive NO_3_^−^ fertilization has become a major environmental concern worldwide, contributing to the production of nitrous oxide (N_2_O), an important greenhouse gas [1,2]. Also, large amounts of non-used fertilizers, including NO_3_^−^, end up in aquatic ecosystems and aquifers, leading to eutrophication, pollution, and human health issues [2,3,4,5]. Therefore, intense efforts are devoted to promoting sustainable agriculture so as to reduce inorganic N inputs and enhance plant N use efficiency. Because N is a central element in various metabolic pathways, it plays a central role not only in plant growth and development, but also in plant/pathogen interactions and plant defense responses [6,7]. In fact, primary N metabolism provides the material to synthesize plant defense compounds and involves remobilization of N-containing compounds, preventing their consumption by pathogens [7,8]. For example, pathogen attacks have been shown to impact on several plant N metabolites such as γ-aminobutyric acid (GABA) or polyamines (PAs) [9,10]. Also, N nutrition has been found to change the susceptibility of *Arabidopsis* rosettes to the necrotrophic fungus *Alternaria brassicicola*, along with a modification of the spectrum of major amino acids [11]. PAs like putrescine, spermidine and spermine have often been more abundant in plants subjected to biotic and abiotic stresses [12,13,14,15,16]. They are known to be associated with plant defense and the interplay between PAs and N nutrition has been proposed to play a role in plant response to abiotic and biotic stresses [17].

Several plant specialized metabolites also play a major role in plant defense and immunity, in particular alkaloids, isoprenoids, and phenylpropanoids [18]. Many phytoalexins are phenylalanine-derived phenylpropanoids [19]. Alkaloids, which are nitrogenous compounds mostly synthesized via the citrate cycle or shikimate pathway [20], include the well-known tryptophan derivative camalexin, involved in *Arabidopsis* resistance to a broad spectrum of pests and diseases [21,22,23]—including *A. brassicicola* [24]. Also, indole glucosinolates (IGS) derive from aromatic amino acids such as tryptophan or phenylalanine [22,25], and are important defense compounds in Brassicaceae. For example, they are involved in the *Arabidopsis*-*A. brassicicola* pathosystem [26]. IGS-based defense relies on the breakdown of IGS by myrosinases, producing a range of chemically active compounds that are toxic to herbivores and pathogens [27].

N fertilization has various effects on disease development, promoting either the host or the pathogen [6,7,28], and therefore, no general rule can be drawn. It is believed that the influence of plant N nutrition on infection outcome depends on the pathogenic lifestyle (biotrophs and hemitrophs vs. necrotrophs) [28,29]. However, it also depends on plant species/pathogen combinations, and even on plant cultivar/pathogen strain combinations [30,31,32]. The specific effects of the two main forms of N supply [i.e., NO_3_^−^ or ammonium (NH_4_^+^)] on pathogen susceptibility and symptoms vary broadly and can even be opposite depending on host–pathogen systems considered [11,33,34,35,36,37]. Presumably, such a variability is linked to differences in metabolic pathways associated with NO_3_^−^ and NH_4_^+^ utilization, both in the plant and in the pathogen [6,38].

The current literature on the influence of N fertilization on plant/pathogen interactions deals with adult plants or seedlings at late developmental stages [28,29,35,39,40]. There is presently a lack of knowledge on the impact of N nutrition on disease development at early stages. This is potentially problematic since current agricultural practices (limitation of inorganic N fertilizer application, reduction in fungicide seed treatments) should promote seed-borne pathogens such as necrotrophic fungi. In other words, there is considerable uncertainty as to whether N plant nutrition modulates fungal disease during very early stages of plant development.

We have previously demonstrated that N nutrition modifies the susceptibility of *Arabidopsis* to *A. brassicicola* and the effect of N nutrition depends on plant developmental stage [11,41]. Adult plants (rosettes) are less susceptible to infection when grown with NH_4_^+^ compared to NO_3_^−^ [11], while seedlings grown with NH_4_^+^ or at low (0.1 mM) NO_3_^−^ concentration are more susceptible to the fungus compared to NO_3_^−^-grown (5 mM) seedlings [41]. In other words, there is a strong interaction between N nutrition and the development stage in this pathosystem. However, the metabolic origin of such an interaction is not known. This aspect is potentially important since it could allow the identification of specific metabolites that inhibit or stimulate fungus growth. Here, we looked at the effect of N nutrition on seedling metabolism and its response to fungal infection, using the same pathosystem (*Arabidopsis*-*A. brassicicola*), with two *Arabidopsis* ecotypes (Col-0 and L*er*) that have contrasting susceptibility to *A. brassicicola*. We looked at germination kinetics, seedling development, ion contents (including nitrate and ammonium), and metabolome (using GC-MS profiling). We used multivariate statistics to identify metabolites that are best correlated to disease symptoms (quantified via the proportion of affected green surface area).

## 2. Results

### 2.1. Impact of Inoculation and Nitrogen Conditions on Germination and Shoot Development

#### 2.1.1. Seed Germination

**Figure 1:**
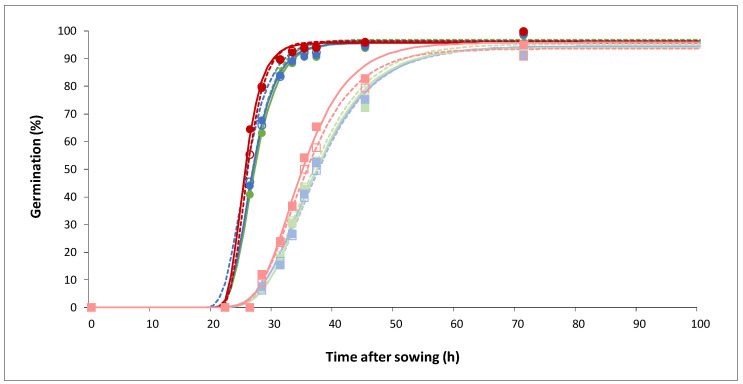
Simulated germination curves (according to the Gompertz model) of seeds of two genotypes (Col-0: dark line and round dots; L*er*: light line and square dots) in three N nutritive media (0.1 mM NO_3_^−^: green line; 5 mM NO_3_^−^: blue line; 5 mM NH_4_^+^: red line), in the presence (Abra43: dotted line and empty dot) or absence (H_2_O: solid line and full dot) of the fungus. The dots correspond to the mean of the experimental values. n = 6; three biological replicates composed of two technical replicates of approximately 70 seeds per set of conditions.

Seed germination was followed in Petri dishes containing three N nutritive media, 0.1 mM NO_3_^−^ as a control (i.e., minimum N content in soils), and 5 mM NO_3_^−^ vs. 5 mM NH_4_^+^. Seeds were inoculated either with *A. brassicicola* (Abra43, referred to as I) or treated with H_2_O (seeds not inoculated with the fungus, NI). Seed germination differed significantly between genotypes (“G” effect), germination being faster in Col-0 (average T50 of 26.4 h), and slower in L*er* (average T50 of 36.5 h), without any impact of inoculation (“I” effect) (Figure 1; Table S1). A significant effect of N conditions (“N” effect) was found, with seeds germinating more rapidly under 5 mM NH_4_^+^ (average T50 of 30.4 h) than the two other media (average T50 of 32 h). No interaction effect was found (Table S1). Differences between genotypes and N conditions observed for T50 were also found throughout germination time courses (Figure 1). The final percentage of germination was very high in both genotypes under all conditions (97–100% at 100 h after sowing), although they were significantly higher for Col-0 (99.8%) compared to L*er* (98%) (Figure 1; Table S1).

#### 2.1.2. Seedling Aerial Development

**Figure 2:**
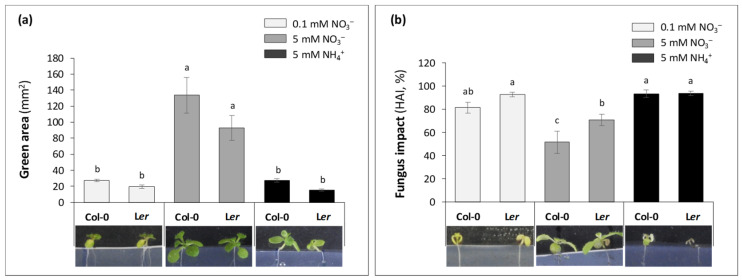
Seedling aerial development for Col-0 and L*er* seedlings grown on different nutritive media (14 DAI). (**a**) Green area (per square plate) in the absence of the fungus (seeds treated with sterile H_2_O). (**b**) Green area ratio (in %) between seedlings from seeds treated with H_2_O and those from seeds inoculated with Abra43 (healthy area index—HAI). The green areas were quantified using a method based on a semi-automated image analysis pipeline [41]. Pictures are representative of seedlings, for each growth condition. Twelve seedlings per square plate, 6 square plates per independent experiment, 3 independent experiments. Error bars indicate SEM. Letters denote significant differences at the 0.05 level.

Based on results found in [41], seedlings were grown for 14 days after seed inoculation (DAI) with Abra43 or H_2_O in square plates (12 seeds sown per plate) containing three N nutritive media (0.1 mM and 5 mM NO_3_^−^, and 5 mM NH_4_^+^). Shoot development was monitored with the green surface area and the healthy area index (HAI, corresponding to the green surface area reduction (in %) in seedlings from seeds inoculated with Abra43 compared to seeds treated with H_2_O) (Figure 2; Table S2). As expected, in the absence of fungus, the green area was significantly higher under 5 mM NO_3_^−^ nutrition compared to other N conditions (Figure 2a; Table S2a). Significant differences were also found between genotypes, with Col-0 showing higher green area (62.7 mm^2^ in average) than L*er* (42.7 mm^2^), whereas G × N was not significant. Regarding HAI, G and N effects were also significant and G × N not significant (Table S2b). In line with our previous results, the highest NO_3_^−^ availability was associated with the best seedling resistance [41]. Aerial development of seedlings grown under 5 mM NH_4_^+^ and 0.1 mM NO_3_^−^ was dramatically affected by infection, HAI being as high as 87%. That is, despite a 61% reduction in green area, seedlings grown under 5 mM NO_3_^−^ were significantly less affected compared to other nutritive media. Interestingly, Col-0 seedlings were less affected by infection under NO_3_^−^ conditions (both at 5 mM and 0.1 mM) than L*er*, while seedlings of both genotypes were similarly affected by infection when grown with NH_4_^+^.

### 2.2. Impact of Inoculation and Nitrogen Nutrition on Ions

**Figure 3:**
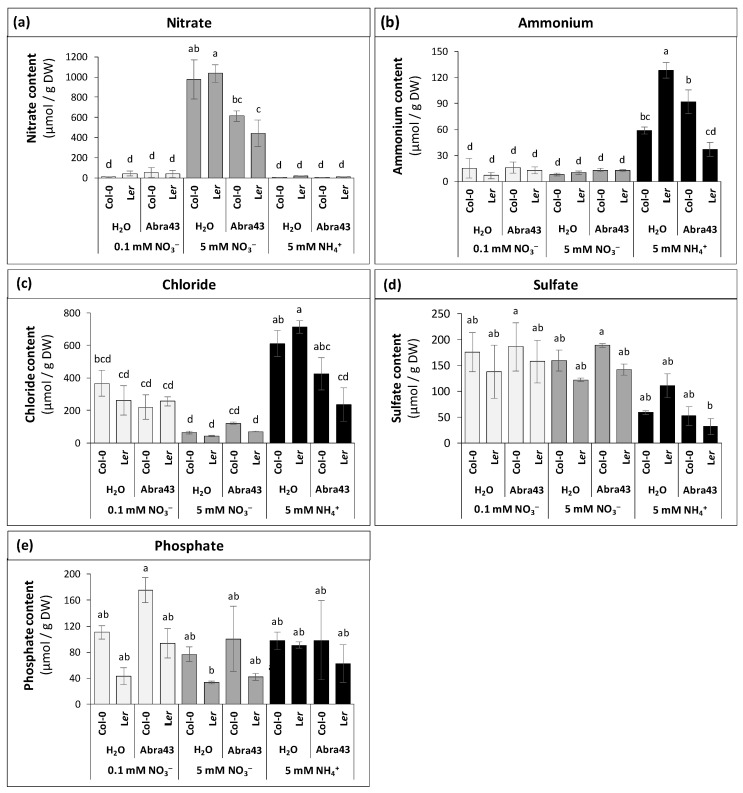
Ion contents of Col-0 and L*er* seedlings grown on different nutritive media (0.1 mM NO_3_^−^, 5 mM NO_3_^−^ or 5 mM NH_4_^+^) from seeds treated with sterile H_2_O or inoculated with Abra43 (14 DAI). (**a**) nitrate, (**b**) ammonium, (**c**) chloride, (**d**) sulphate, and (**e**) phosphate contents in seedlings grown on different N nutrition and inoculation conditions. Error bars indicate SEM (n = 3 biological replicates). Letters denote significant differences at the 0.05 level.

As expected, the nitrate content differed between N nutrition conditions, with more nitrate in tissues under 5 mM NO_3_^−^ (Figure 3a). The nitrate content also differed between inoculation conditions, with an interaction between the two factors (Figure 3a; Table S3). In effect, inoculation by the fungus led to a decline in nitrate content in both genotypes, in particular in L*er* (Figure 3a). Ammonium content was affected by N conditions, and several interaction effects were significant, N × I, I × G and N × I × G (Figure 3b; Table S3). Unsurprisingly, seedlings grown under 5 mM NH_4_^+^ were the richest in ammonium. Interactions between factors were mainly observed under 5 mM NH_4_^+^ condition where infection modified the ammonium content depending on genotype (increased in Col-0, decreased in L*er*). As a result, the ammonium content in Abra43-infected seedlings was higher in Col-0 compared to L*er*.

N conditions affected the chloride content, which was higher under ammonium nutrition (Figure 3c; Table S3). Inoculation also influenced the chloride content, and the N × I interaction was significant, mostly explained by the decrease in chloride at 5 mM NH_4_^+^ in Abra43-inoculated seedlings. Such a decrease was more pronounced in L*er* seedlings. The sulphate content was only influenced by N nutrition (Figure 3d; Table S3), while phosphate was only affected by genotype (Figure 3e; Table S3), with Col-0 seedlings being on average richer in phosphate than L*er* seedlings.

### 2.3. Effect of Genotype, N Nutrition, and Inoculation Conditions on Seedling Metabolome

**Figure 4:**
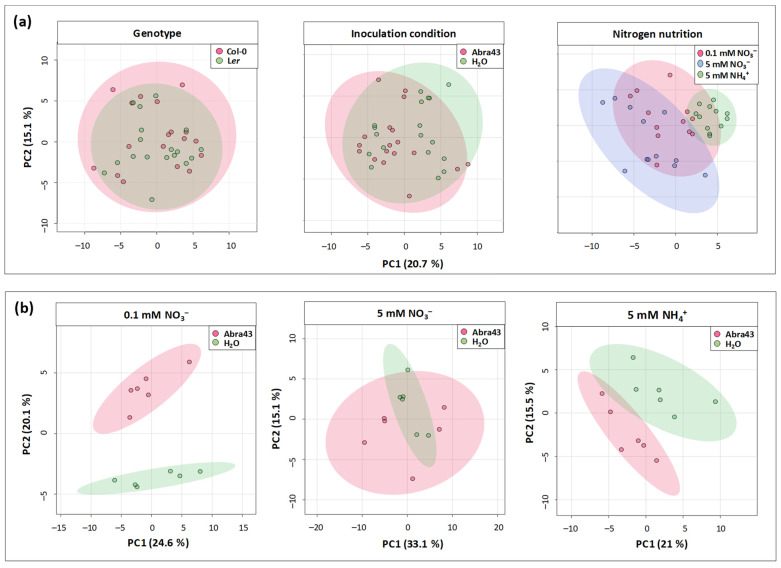
Principal component analysis (PCA) of total metabolite contents (amino and organic acids, sugars, and polyamines) of 14 days seedlings. (**a**) Separation of samples according to the three experimental factors studied (**top**). (**b**) Separation of samples treated either with sterile H_2_O or inoculated with the fungus (Abra43) according to N nutrition (**bottom**). The factors were genotype [Col-0; L*er*], inoculation condition [seeds treated with sterile H_2_O; seeds inoculated with Abra43], nitrogen nutrition [0.1 mM NO_3_^−^; 5 mM NO_3_^−^; 5 mM NH_4_^+^]. Each point represents a sample, corresponding to the metabolite contents in one condition of genotype, inoculation, and N nutrition. n = 3 biological replicates.

Metabolomics analyses were carried out on seedlings collected 14 days after seed sowing, using GC-MS. A first principal component analysis (PCA) was performed to examine the main factors discriminating samples, via a separation of metabolic contents per condition of genotype, inoculation, and N nutrition (Figure 4a). The two first principal components (PC) explained 35.8% of the variance, with PC1 and PC2 explaining 20.7% and 15.1% (Figure 4a). The samples were only separated based on N conditions, the 0.1 mM NO_3_^−^ group being intermediate. It thus appears that the impact of N conditions on metabolite contents was greater than that of inoculation and genotype. PCAs were then performed to assess sample grouping according to inoculation conditions with each N medium taken separately. The two first PCs explained between 36.5% and 48.2% of variance (Figure 4b). A clear separation of Abra43 and H_2_O samples was found for both 5 mM NH_4_^+^ and 0.1 mM NO_3_^−^ media, but not in the case of 5 mM NO_3_^−^ medium (Figure 4b). Taken as a whole, there was an influence of N nutrition on the metabolome, and the effect of inoculation was only visible under conditions where seedlings were more affected to the fungus (i.e., 5 mM NH_4_^+^ and 0.1 mM NO_3_^−^), regardless of the genotype.

### 2.4. Metabolites Affected by Inoculation and Nitrogen Nutrition

#### 2.4.1. Univariate Analysis

**Figure 5:**
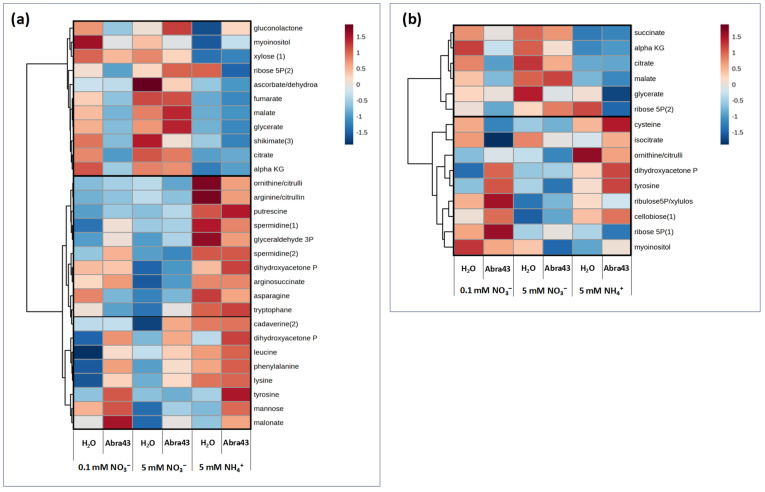
Metabolomic patterns of seedlings grown under different N nutritional media (0.1 mM and 5 mM NO_3_^−^, 5 mM NH_4_^+^), from seeds treated with H_2_O or inoculated with the fungus (Abra43). (**a**) Heatmap showing metabolites presenting significant differential abundances between N media and inoculation conditions (*p* < 0.05; ANOVA followed by Tukey’s HSD test) with a hierarchical clustering. (**b**) Heatmap showing metabolites presenting a significant N medium × inoculation interaction effect. Data from the two genotypes were pooled. The metabolite contents were log-transformed and the values were centered on the median value. A red color corresponds to metabolites more abundant than the median, and a blue color to metabolites less abundant than the median. Clustering of metabolites was established by Ward’s method, with a measure of the Euclidean distance. Numbers after metabolite names (i.e., DHAP(1)) correspond to different derivation products associated with the same molecule.

Since there was little genotype effect on metabolome under our conditions, the analysis of variance (two-way ANOVA) with hierarchical clustering was carried out with pooled genotype data. In the first heatmap (Figure 5a), metabolites associated with a significant N effect or a significant I effect are shown. Two main groups of metabolites were observed. The first group comprised 11 metabolites generally more abundant in seedlings without inoculation in the presence of nitrate. They were also more abundant in inoculated seedlings grown on 5 mM NO_3_^−^. These metabolites were organic acids of the tricarboxylic acid pathway (TCAP; like fumarate, malate, citrate, and 2-oxoglutarate), glycerate, shikimate, and sugar derivatives (gluconolactone, myoinositol, xylose, ribose 5-phosphate, and (dehydro)ascorbate). The second group was formed by two main sub-clusters. The first sub-cluster comprised metabolites less abundant under 5 mM NO_3_^−^ and more abundant under 5 mM NH_4_^+^ such as amino acids (including asparagine and tryptophan), urea cycle intermediates (arginine, citrulline/ornithine, arginine-succinate) and their derivatives (cadaverine, putrescine, and spermidine), and triose-phosphates. The second sub-cluster comprised seven metabolites that were more abundant in inoculated seedlings in particular under 5 mM NH_4_^+^, such as amino acids (leucine, lysine, phenylalanine, tyrosine), mannose, malonate, and triose-phosphates.

The second heatmap (Figure 5b) shows metabolites associated with a significant N x I effect. Two groups were visible. The first group comprised six metabolites, such as organic acids (malate, glycerate, citrate, and 2-oxoglutarate, succinate), and ribose 5-phosphate. These metabolites were more abundant both in the absence of the pathogen with nitrate, and in infected seedlings under 5 mM NO_3_^−^. The second cluster comprised metabolites with no clear pattern, although they were generally more abundant in inoculated seedlings under the two N conditions where seedlings were more affected by the fungus (1 mM NO_3_^−^ and 5 mM NH_4_^+^). They were either amino acids (cysteine, tyrosine), urea cycle intermediate (citrulline/ornithine), organic acid (isocitrate), or sugar derivatives (triose-phosphates, ribulose/xylulose 5-phosphate, ribose 5-phosphate, cellobiose, myoinositol).

#### 2.4.2. Supervised Multivariate Analysis (OPLS)

**Figure 6:**
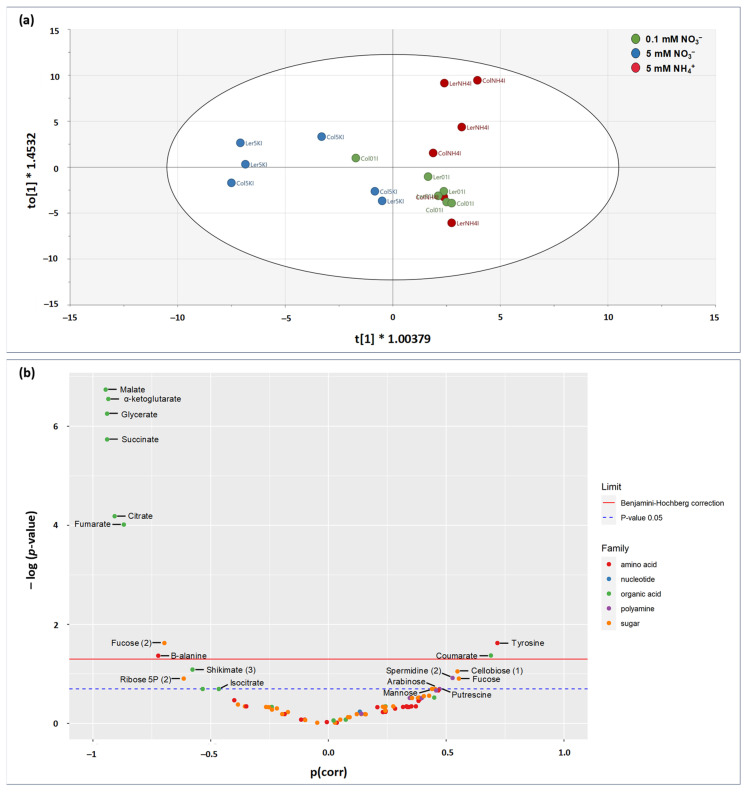
Relationship between metabolites determined by GC-MS analysis (amino acids, organic acids, polyamines, and sugars) and fungus quantitative impact on seedlings determined by green area ratio index (HAI: % of green area between seedlings from seeds treated with H_2_O and those from seeds inoculated with Abra43). (**a**) Score plot of the multivariate analysis by OPLS (Orthogonal Projections to Latent Structures) demonstrating the good sample discrimination according to HAI (horizontal axis). Samples represent the determination of metabolites under a given condition of genotype, inoculation, and N nutrition. Data were mean-centered. In the score plot, the samples from the 5 mM NO_3_^−^, 0.1 mM NO_3_^−^, and 5 mM NH_4_^+^ nutrition are represented in blue, green and orange, respectively. (**b**) Volcano plot showing the best discriminating metabolites, with the *y*-axis corresponding to -log of the *p*-value (adjusted with the Benjamini–Hochberg method) of the linear regression of the HAI on the content of a given metabolite in each experimental condition, and the *x*-axis corresponding to the loading in the OPLS (p(corr)). The dotted blue line corresponds to -log of the *p*-value at the 0.05 threshold, and the red line corresponds to this *p*-value after correction by the Benjamini–Hochberg method. Significant metabolites on the left are associated with the lowest HAI, and those on the right are associated with the highest HAI. The numbers after the metabolite names (i.e., fucose (1)) correspond to different derivation products associated with the same molecule.

To identify metabolites specifically associated with the impact of the fungus on aerial seedling development, a supervised multivariate analysis was performed using quantitative orthogonal partial least square (OPLS) (Figure 6a and Figure S1), where metabolites were used as predictive (X variables) and HAI was used as the response Y variable (R^2^ = (0.0, 0.769), Q^2^ = (0.0, −0.649), and Pcv-anova = 0.017). Unsurprisingly, the score plot differentiated samples according to N conditions along axis 1, simply because of the effect of N nutrition conditions on the susceptibility to the fungus.

The contribution of metabolites in the multivariate relationship to HAI was explored using a volcano plot (Figure 6b) which shows the *p*-value from univariate regression analysis (*y*-axis) as a function of the loading in the OPLS (p(corr), *x*-axis). In such a representation, significant metabolites on left are associated with low HAI values, while metabolites on right are associated with high HAI values. Low HAI-associated metabolites were organic acids of the TCAP (malate, 2-oxoglutarate, succinate, citrate, fumarate, isocitrate), glycerate, shikimate, sugars (fucose, ribose 5-phosphate), as well as the non-proteinogenic amino acid β-alanine. High HAI-associated metabolites were coumarate and tyrosine (two metabolites of the phenylpropanoid pathway), along with sugars of hemicelluloses (mannose, arabinose, cellobiose, and fucose) and polyamines (spermidine, putrescine).

### 2.5. Transcript Abundance of Selected Genes

**Figure 7:**
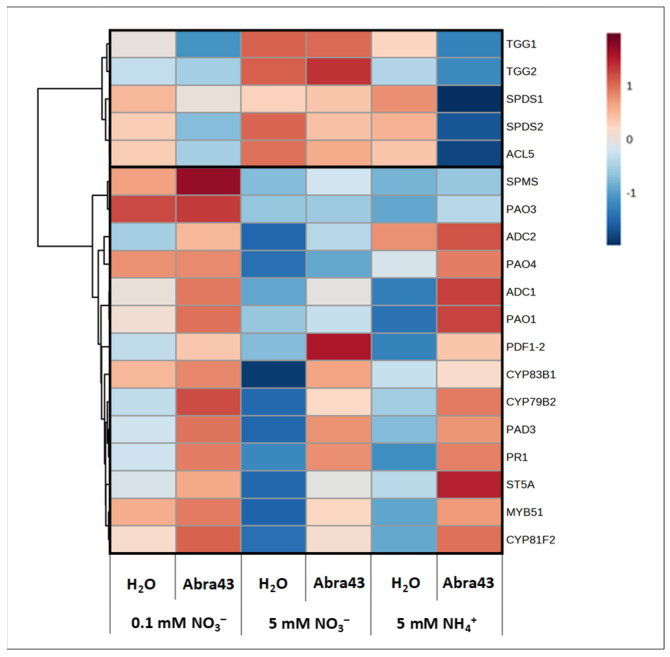
Transcript patterns of selected genes expressed in seedlings grown under different N nutritional media (0.1 mM and 5 mM NO_3_^−^, 5 mM NH_4_^+^), from seeds treated with H_2_O or inoculated with the fungus (Abra43). Heatmap showing transcript patterns of selected genes presenting significant differential expression between N media and inoculation conditions (*p* < 0.05; ANOVA followed by Tukey’s HSD test) with a hierarchical clustering. Data from the two genotypes were pooled. The expression value of the genes (obtained using the following formula: 2^−(CT gene of interest—CT mean of the two reference genes)^) were log-transformed, and the values were centered on the median value. A red color corresponds to genes more expressed than the median, and a blue color to genes less expressed than the median. The selected genes are involved in plant defense (*PDF1.2*, P*R1*), in synthesis of defense compounds derived from tryptophan (*CYP79B2*, *PAD3*, *CYP83B1*, *MYB51*, *ST5A*, *CYP81F2*), in myrosinase synthesis (*TGG1*, *TGG2*), and in polyamine synthesis (*ADC1*, *ADC2*, *SPDS1*, *SPDS2*, *ACL5*, *SPMS*) and oxidation (*PAO1*, *PAO3*, *PAO4*).

Transcript accumulation of selected genes involved in defense mechanisms was assessed via quantitative RT-PCR. We deliberately chose genes of specialized metabolism, based on results of metabolomics (polyamines, glucosinolates; see detailed list in Table S4). An analysis of variance (two-way ANOVA) was undertaken to identify transcripts associated with significant N and I effects (with pooled genotype data; Figure 7). Hierarchical clustering of significant genes forms two groups. The first cluster comprised five genes associated with polyamine biosynthesis (spermidine synthase genes, *SPDS1* and *SPDS2*, and thermospermine synthase gene, *ACL5*), as well as the genes involved in glucosinolate breakdown, encoding myrosinases, *TGG1* and *TGG2*. These genes were down-regulated by inoculation under NH_4_^+^ or low NO_3_^−^ but not under 5 mM NO_3_^−^ (i.e., the condition under which seedlings were less affected). The second cluster comprised genes with specific inoculation effects: (*i*) induced in inoculated seedlings regardless of N conditions (*PDF1.2* and *PR1*, and genes of tryptophan-derived specialized metabolism: *CYP79B2*, *CYP83B1*, *MYB51*, *ST5A*, *CYP81F2*, and *PAD3*), (*ii*) not induced in inoculated seedlings under 5 mM NO_3_^−^ (genes of polyamine metabolism: *SPMS* and *PAO1* and *4*, *ADC1* and *2*). Taken as a whole, 5 mM NO_3_^−^ led to specific expression patterns, in particular of polyamine metabolism, while other genes responded to inoculation (biosynthesis of camalexin and IGS) regardless of N supply conditions, suggesting that the modest lower effect of inoculation under high NO_3_^−^ was linked to the reconfiguration of polyamine metabolism.

## 3. Discussion

### 3.1. Beneficial Effect of High Nitrate Conditions on Seedling Susceptibility

We previously showed that *Arabidopsis* susceptibility to the fungal pathogen *A. brassicicola* depended on both N nutrition and development stage (seedling vs. adult) [11,41]. In effect, seedlings grown under NO_3_^−^ nutrition (5 mM) were less susceptible compared to NH_4_^+^ nutrition (5 mM), whereas the opposite effect of N nutrition was observed in rosettes. Here, we focused on seed germination and early seedling growth (Figure 1 and Figure 2). Our results clearly show the advantage of NO_3_^−^ nutrition for seedling aerial development (regardless of pathogen presence), and also that Col-0 has some advantage compared to L*er* because it germinates and develops faster. Also, under 5 mM NO_3_^−^, the impact of the pathogen was significantly lower (Figure 2). This beneficial effect of NO_3_^−^ was not linked to a defect in inoculation since some defense genes were effectively activated (Figure 7) and there were visible symptoms of infection on seedlings (Figure 2) including under 5 mM NO_3_^−^. That is, our result suggests that there is a lower susceptibility of seedling to *A. brassicicola* under higher NO_3_^−^ likely due to nutritive (metabolic) and signaling mechanisms, which in turn modulate growth and developmental processes in *Arabidopsis* in a way that is detrimental to the pathogen [42].

### 3.2. Metabolism Involved in the Generic Response to A. brassicicola

Under our conditions, several metabolites were associated with a significant I effect, showing that seedlings responded to inoculation via the regulation of several metabolic pathways (Figure 5). This included amino acids (leucine, lysine, phenylalanine, tyrosine) and other metabolites (mannose, dihydroxyacetone phosphate, malonate) which were more abundant in inoculated seedling regardless of N conditions (Figure 5). Such changes are likely reflective of the induction of glucosinolate metabolism, which are synthesized from amino acids (alanine, leucine, isoleucine, phenylalanine, tryptophan, tyrosine, and valine), with methionine (or cysteine via trans-sulphuration) as the S-atom (thiol) donor. Indole glucosinolates (IGS) are derived from aromatic amino acids such as tryptophan or phenylalanine [22,25]. Accordingly, genes associated with glucosinolate metabolism (in particular tryptophan-derived glucosinolates) were found to be induced by inoculation (Figure 7).

When the statistical analysis was carried out using HAI (regardless of N conditions), many metabolites appeared to be linked to the response to inoculation (Figure 6). In particular, there was a general decrease in organic acids of the TCAP, and an increase in cell-wall-associated sugars, phenylpropanoids (couramate), and the polyamine spermidine. Effects on cell wall biochemistry are expected in plant/pathogen interactions [43,44], in particular with necrotrophic and hemi-biotrophic pathogens which secrete cell wall hydrolytic enzymes [45,46]. The effect on polyamine is consistent with other studies [10,17]. Polyamines tend to increase in infected tissues during microbial colonization, independently of the nature of the pathogen (biotroph or necrotroph) [47,48]. Jubault et al. [49] have also reported that in the *Arabidopsis*-*Plasmodiophora brassicae* pathosystem, susceptible plants displayed a transient accumulation of agmatine (putrescine precursor) and strong arginase activity (which cleaves arginine into ornithine), whereas partially resistant plants showed continuous agmatine production and weak arginase activity.

The phenylpropanoid pathway is at the origin of flavonoids, lignin, but also salicylic acid (SA), which is involved in plant defense [50]. SA is generally associated with induced resistance against biotrophic pathogens whereas ethylene and jasmonic acid (JA) pathways induce resistance against necrotrophic pathogens, with an antagonism SA–JA signaling crosstalk [51,52,53]. Interestingly, the gene expression of *PDF1.2*, involved in JA synthesis, was lower under 0.1 mM NO_3_^−^ and 5 mM NH_4_^+^ compared to 5 mM NO_3_^−^ in presence of *A. brassicicola* (Figure 7), suggesting a lower JA production would lead to a higher susceptibility.

We nevertheless recognize that part of the relationship between metabolites and HAI in Figure 6b was driven by N conditions, since high nitrate was generally associated with low HAI, while other conditions were associated with high HAI. In particular, the generic effect on organic acids, which are more abundant at low HAI (Figure 5b and Figure 6b), was likely related to a confounding effect of high NO_3_^−^ (as opposed to NH_4_^+^ nutrition). In effect, NO_3_^−^ nutrition usually leads to higher organic acid synthesis, via both the TCAP and anaplerotic fixation by phosphoenolpyruvate carboxylase. That said, in response to infection, it has been shown that the strong demand in energy and carbon backbones could involve amino acids as respiratory substrates [54], generating organic acids. Also, TCAP intermediates have been also suggested to play a role in plant defense: citrate and fumarate have been shown to be able to induce priming in *Arabidopsis* against the bacterial pathogen *Pseudomonas syringae* pv. *tomato* DC3000 [55]. In other words, the content in TCAP intermediates is probably a compromise between energy requirements, signaling and amino acid utilization, which in turn depends on infections by pathogens.

### 3.3. Metabolic Mechanisms of the Effect of N Conditions on Susceptibility

The effect of N nutrition on susceptibility (Section 3.1) was associated with specific metabolites; that is, several metabolites were associated with an interaction effect N x I (Figure 5b). In addition to organic acids (that are partly driven by NO_3_^−^ assimilation itself, see above), some metabolites of various pathways accumulated less or even decreased upon inoculation under high NO_3_^−^ (5 mM). They included metabolites reflecting defense response to pathogens, such as tyrosine, polyamine precursors (ornithine/citrulline) or sugars (cellobiose, myoinositol). The specific effect on polyamine metabolism was corroborated by transcript analysis, which suggested a specific regulation under 5 mM NO_3_- (Figure 7). Under high NO_3_^−^, genes associated with spermidine (*SPDS*) and thermospermine (*ACL5*) synthesis are more expressed. Also, there is a low expression level and little response to inoculation of genes encoding polyamine oxidases (*PAO*), arginine decarboxylases (*ADC*), and spermine synthase (*SPMS*). In addition to the specific role of polyamines on plant–pathogen interaction itself, the turn-over of polyamines via PAO generates hydrogen peroxide (H_2_O_2_) that can participate in defense responses. It is thus plausible that high NO_3_^−^ down-regulates the effect of inoculation via polyamine catabolism.

The specific effect of high NO_3_^−^ on tyrosine (decrease upon inoculation, unlike under low NO_3_^−^ or NH_4_^+^) was perhaps explained by increased utilization of its precursors (chorismate, shikimate). Genes encoding enzymes of the tryptophan-derived specialized metabolic defense pathways (*CYP79B*, *CYP83B1*, *MYB51*, *ST5A*, *CYP81F2*, and *PAD3*) were induced considerably in inoculated seedling (Figure 7). Concurrently, basal level of myrosinase gene expression (*TGG*) was elevated under high NO_3_^−^ conditions, suggesting intense indole glucosinolates (IGS) metabolism [22,25]. In other words, there was perhaps a competition for chorismate utilization, in favor of tryptophan synthesis and at the expense of tyrosine production under 5 mM NO_3_^−^. This would be consistent with the fact that a precursor of shikimate (chorismate) synthesis, ribose 5-phosphate, increased upon inoculation under high NO_3_^−^, while the opposite was observed under low NO_3_^−^ or NH_4_^+^ (Figure 5b).

In summary, the beneficial effects of 5 mM NO_3_^−^ on seedling subjected to *A. brassicicola* inoculation may be originated from a constitutively high tryptophan metabolism, along with a down-regulation of oxidative stress caused by polyamine catabolism. We note that a common point shared by polyamine and glucosinolate metabolism is the involvement of sulfur assimilation and metabolism, since polyamine biosynthesis requires S-adenosylmethionine and glucosinolate biosynthesis require methionine and/or cysteine. Interestingly, under NO_3_^−^ nutrition, the cysteine content declined, and the sulphate content did not change upon inoculation (Figure 3 and Figure 5b). Opposite variations were observed under NH_4_^+^ nutrition, where symptoms after inoculation (HAI) were maximal. Thus, it is also possible that sulfur homeostasis is involved in the specific response to the pathogen under NO_3_^−^ nutrition.

## 4. Materials and Methods

### 4.1. Plant and Fungal Materials

Two ecotypes of *A. thaliana* (Col-0 and L*er*) were analyzed. Seeds of each genotype were obtained from plants grown for 13 weeks under long-day conditions (16 h of light at 21 °C, 8 h of darkness at 19 °C) in a growth chamber (IRHS, Angers, France). The Abra43 wild-type strain of *A. brassicicola* was isolated from *Raphanus sativus* seeds [24], and sequenced [56]. It was grown and maintained on potato dextrose agar at 24 °C.

### 4.2. Experimental Device Developed for Arabidopsis Seedling Growth

According to the method developed by Barrit et al. [41], seedlings of *A. thaliana* were grown vertically in square plates (12 × 12 × 1.3 cm) filled with 1.2% agar modified MS medium (0.5 mM CaCl_2_, 0.5 mM MgSO_4_, 1 mM KH_2_PO_4_, 50 µM iron-EDTA, and 0.5 mL/L micro-elements buffered with 0.5 g/L MES (2-(N-morpholino)ethanesulfonic acid), pH 5.7) [57], supplemented either with 5 mM NO_3_^−^ (provided as KNO_3_), 0.1 mM NO_3_^−^ or 5 mM NH_4_^+^ (provided as NH_4_Cl). For each plate, 12 seeds were sown on a plane surface obtained by cutting the medium 3 cm from the top. Seeds were stratified for 3 days before sowing in order to break their residual dormancy. They were then sterilized by successive immersions (5 min in ethanol 70°, 15 min in sodium hypochlorite (2.6% active chlorine) and three times 5 min in sterile water). Sown seeds were individually inoculated by deposit of a 1 µL droplet of conidia suspension (10^4^ conidia per mL; Abra43 condition) or of sterile milli-Q water (H_2_O condition). The plates were then placed in a growth chamber under long-day conditions (16 h of light at 21 °C, 8 h of darkness at 19 °C) for 14 days. Three independent experiments were carried out. Each experiment included 50 plates of 12 seedlings per set of conditions.

### 4.3. Seed Germination

Seed germination was specifically studied by sowing seeds of the two genotypes in 50 mm Petri dishes, using the same media, inoculation method and growing conditions than for seedling growth experiments. Three biological replicates were performed, each with two technical replicates of 50–100 seeds. Germination was followed up to 144 h after sowing.

### 4.4. Image Analysis

Six pictures of square plates containing seedlings were taken, per set of conditions and per independent experiment (to a total of 72 pictures per independent experiment), using a Nikon D5000 digital camera (Nikon, Tokyo, Japan), in the same lighting and distance conditions. Image analysis was performed according to the protocol established in Barrit et al. [41]. The classification of images was carried out with the Ilastik software (version 1.3.3) [58]. The training dataset was annotated by associating three classes of pixels defining to healthy tissues (green), necrotic tissues (brown), and all other objects present in images that do not correspond to aerial parts of seedlings. Once the model had been trained, it was applied to the other images to identify the healthy (green) and necrotic (brown) pixels of aerial seedling parts (leaves, cotyledons, and hypocotyl) in each square plate. Classification results were then subjected to a Python 3.7 script to evaluate healthy and necrotic tissue areas and generate the color-coded final images. This script was based mainly on the numpy [59], *scikit-image* [60], and matplotlib [61] libraries. The healthy area index (HAI), defined by Barrit et al. [41], was then used to evaluate the impact of the fungus on seedling development. It depicts the percentage of healthy area variation between seedlings treated with H_2_O and Abra43 and integrates both direct and indirect fungal effects (area deficit due to necrotic tissues and seedling growth deficit, respectively).

HAI = ((HA_H2O_ − HA_Abra43_)/HA_H2O_) × 100 (1)

### 4.5. Metabolite Profiling on Seedlings

Samples of the aerial parts of the seedlings used for all the experiments were collected 14 days after sowing and inoculation, and stored at −80 °C, from the 3 independent experiments carried out.

#### 4.5.1. Inorganic Anions and Ammonium Contents

Samples (50 mg of dry weight) were lyophilized and ground with glass beads (3 mm) using a TissueLyser (Qiagen, Hilden, Germany) at 30 Hz for 1 min. Then, polar metabolites were extracted with 400 μL methanol/1 mL chloroform/400 μL water. Samples were dried using a speed vac (Genevac, Ipswich, UK) and then solubilized in 1 mL deionized water.

For the quantitation of anion contents, 150 μL of sample were analyzed by high-performance liquid chromatography (HPLC), with 12 mM NaOH on an AS11-HC hydroxide-selective-anion-exchange column (Dionex Corporation, Sunnyvale, CA, USA) using conductimetric detection, following the product manual for IonPac AS11 (Thermo Scientific, Waltham, MA, USA). Metabolic data were normalized to dry weight, and three independent experiments were carried out.

A colorimetric method was used to measure ammonium content of seedlings, according to Vega-Mas et al. [62]. An amount of 50 μL of the samples was mixed in a flat-bottom microplate (greiner 96 well), with, in the following order, 100 μL of sodium phenoxide (330 mM) dissolved in NaOH (160 mM, pH ≈ 13), 50 μL of sodium nitroprusside (0.02%), and 100 μL of sodium hypochlorite (0.2%). After 30 min incubation at room temperature, absorbance was read at 635 nm. A standard curve was prepared from a 10 mM ammonium sulphate solution to determine the NH_4_^+^ content. Three independent experiments were carried out in duplicate.

#### 4.5.2. GC-MS-Based Targeted Metabolomics

Gas chromatography-mass spectrometry was performed using a 436-GC coupled to a Simple Quadruple (SQ) SCION MS (Bruker^®^, Bremen, Germany). The column was an RTX-5 w/integra-Guard (30 m × 0.25 mm i.d. + 10 m integrated guard column; Restek). Samples were ground in a mortar in liquid N_2_ (30 mg of fresh powder) and then in 1 mL of 80% methanol, to which ribitol (100 μmol L^−1^) was added as an internal standard. Extracts were transferred to 2 mL Eppendorf tubes and centrifuged at 10,000× *g* and 4 °C for 15 min. Supernatants were dried with SpeedVac and stored at −80 °C. Methoxyamine was dissolved in pyridine at 50 mg mL^−1^, and 40 μL of this mixture was used to dissolve the dry individual samples. After vigorous mixing, samples were incubated at 37 °C with stirring for 90 min. Next, 40 μL of N-methyl-N-(trimethyl-silyl) trifluoroacetamide was added, and the mixture was vortexed and incubated at 37 °C with stirring for 30 min. Before loading into the gas chromatography autosampler, a mixture of a series of fourteen alkanes (chain lengths C10–C36) was included. Analyses were performed by injecting 1 μL in splitless or split mode at 280 °C (injector temperature). Separation was carried out in helium as a carrier gas at a rate of 1 mL min^−1^ in constant flow mode, using a temperature ramp from 70 °C to 320 °C between 4 and 22 min, followed by 5 min at 320 °C. Ionization was reached by electron impact at 70 eV, and the mass spectrum acquisition rate was 20 spectra s^−1^ over the 70–500 m/z range. Peak identity was established by comparing the fragmentation pattern with the mass spectra from our homemade metabolite spectrum database and based on the retention index using the alkane series as retention standards. The peaks were integrated using Bruker^®^ MS Workstation software (version 8).

### 4.6. Gene Expression Profiling by Quantitative Real-Time Reverse Transcription-PCR

For total RNA extraction, seedling aerial parts were ground in liquid nitrogen using a mortar and pestle. The homogenous powder was treated with TRI Reagent (Ambion, Carlsbad, CA, USA) according to the manufacturer’s protocol. After DNAse treatment, cDNAs were obtained by reverse transcription (RT) of 1 μg of total RNA using 6 μL of iScript RT supermix5X (Bio-Rad, Hercules, CA, USA). The reaction mixture was incubated for 5 min for a priming step (25 °C), followed by the RT step (20 min, 46 °C). Quantitative real-time PCR was performed using the SsoAdvanced Universal SYBR Green Supermix (Bio-Rad, Hercules, CA, USA), following the manufacturer’s instructions, and using the CFX96 real-time PCR detection system (Bio-Rad). PCR reactions were incubated for 30 s at 95 °C followed by 40 cycles of 10 s at 95 °C and 10 s at 60 °C. The specificity of the PCR amplification procedure was verified with a heat-dissociation protocol (from 65 to 95 °C) after the final cycle of PCR. Each measurement was performed with three biological repeats, using a triplicate PCR reaction for each modality to determine Ct values. Each gene expression level was normalized with two reference genes (Actin and Ubiquitin), using the following formula: 2^−(CT gene of interest—CT mean of the two reference genes)^. The primer sequences used for quantitative RT-PCR are listed in Table S4.

### 4.7. Statistics

R Software (version 4.2.0) was used to carry out statistical analyses. Parametric tests (analysis of variance [ANOVA] followed by Tukey’s HSD test in the case of multiple comparisons, and Student’s *t* test in the case of additional side-by-side comparisons) were performed when the conditions of normal distribution and homogeneity of variances were met. Otherwise, nonparametric tests (ANOVA using permutation tests for multiple comparisons or Wilcoxon–Mann–Whitney test for additional side-by-side comparisons) were used. *p*-values < 0.05 were considered to be statistically significant. *p*-value calculations for the volcano plot were also performed with the R software (version 4.2.0). MetaboAnalyst 5.0 [63] was used to generate the PCAs and heatmaps, with data normalized with the following procedure: sample normalization by median, log transformation of data, and scaling (mean-centered and divided by the standard deviation of each variable). The hierarchical clustering of metabolites or gene expressions for the heatmaps was established using Ward’s method, with a measure of Euclidean distance. Finally, orthogonal projection on latent structure (OPLS) and p(corr) calculations used in the volcano plot were performed on the SIMCA software version 17.0.2.34594 (Sartorius, Gottingen, Germany), using disease symptoms quantified via the proportion of affected green surface area (HAI) as a quantitative Y variable and metabolic features as predictive variables.

## 5. Conclusions

We have previously shown that there is a strong interaction between N nutrition and development stage (seedling vs. rosette) for Arabidopsis susceptibility to the seed-borne pathogen *A. brassicicola*. Specificity at the seedling stage comes from a beneficial effect of high NO_3_^−^ (5 mM) nutrition compared to low NO_3_^−^ (0.1 mM) or NH_4_^+^ (5 mM) nutrition, which is an opposite profile to that evidenced at the rosette stage. Here, the effect of N nutrition on seedling metabolism and its response to fungal infection suggest that the beneficial effect of high NO_3_^−^ on seedling susceptibility to *A. brassicicola* is probably due to nutrition and signaling mechanisms affecting plant development processes detrimental to the pathogen. The examination of the overall metabolomic profiles and metabolites identified as specifically correlated with high or low disease symptoms (i.e., fungal growth) suggests that it (the beneficial effect of high NO_3_^−^) may originate from a constitutively elevated tryptophan metabolism, as well as down-regulation of oxidative stress caused by polyamine catabolism. It is also possible that sulfur homeostasis is involved in the pathogen-specific response to NO_3_^−^ nutrition. To test these hypotheses, it would be useful to carry out detailed profiling of glucosinolates as a function of seedling N nutrition, as well as a broader metabolic analysis using LC-MS to quantify the players in sulfur metabolism. To go further, it would be interesting to analyze the effect of Arabidopsis N nutrition on *A. brassicicola* growth itself, by infecting seedlings with the fluorescently labelled fungus, and observing colonization and spread under the different N nutrition conditions. The role of some metabolites revealed in the present study (e.g., polyamines) could also be validated by treating seedlings with their precursors or inhibitors, and by observing how they affect disease development.

## Data Availability

All data generated or analyzed during this study are included in this published article and its Supplementary Information File. Script source code used during the current study for image analysis is available from the corresponding author on reasonable request.

## Supplementary Materials

The following supporting information can be downloaded at: https://www.mdpi.com/article/10.3390/plants13040534/s1, Figure S1: supervised multivariate analysis (OPLS); Table S1: seed germination; Table S2: seedling aerial development; Table S3: ammonium and inorganic ion contents; Table S4: list of the primer sequences.
