# Supplementary material for: Nitrogen Nutrition Modulates the Response to Alternaria brassicicola Infection via Metabolic Modifications in Arabidopsis Seedlings
Source: Plants (Basel). 2024 Feb 15;13(4):534. doi: 10.3390/plants13040534 (PMC10892011; doi:10.3390/plants13040534)

Figure S1. **Supervised multivariate analysis (OPLS) demonstrating (a) the determination of genotype in a given condition of inoculation and N nutrition (vertical axis) according to healthy area index (HAI, horizontal axis) and (b) the determination of metabolites (vertical axis) according to HAI (horizontal axis).**

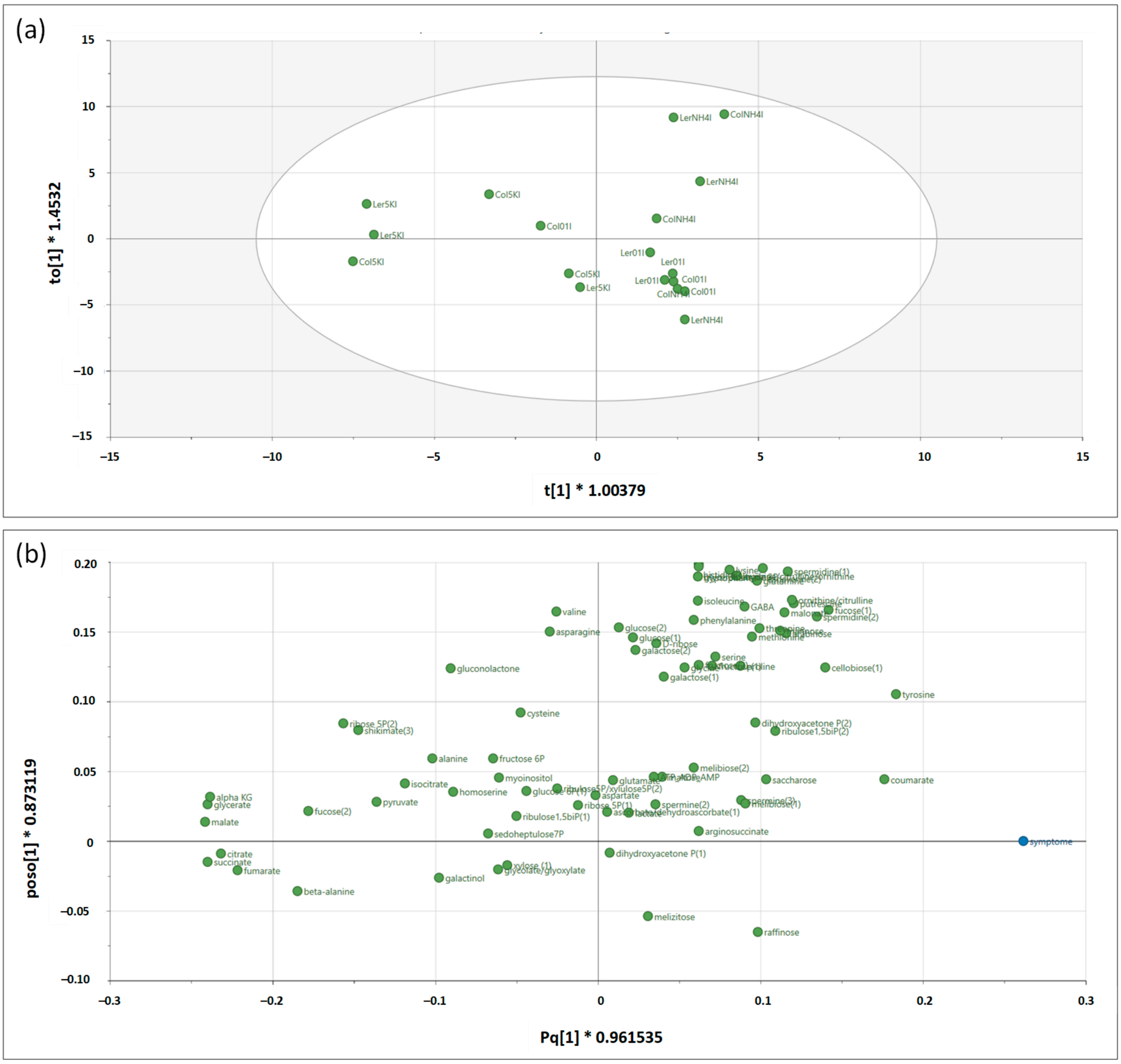

Supplement: Supplementary file 1 [file plants-13-00534-s001.zip › Figure S1.pdf]
