# Supplementary material for: Nitrogen Nutrition Modulates the Response to Alternaria brassicicola Infection via Metabolic Modifications in Arabidopsis Seedlings
Source: Plants (Basel). 2024 Feb 15;13(4):534. doi: 10.3390/plants13040534 (PMC10892011; doi:10.3390/plants13040534)
Supplement: Supplementary file 1 [file plants-13-00534-s001.zip › Table S1.pdf]

Table S1 : Seed germination. (a) Time required for seeds to reach 50% germination (T50 ; in hours) and (b) maximum germination (in %) for seeds of the two genotypes Col-0 and L*er* sown on different nutrient media (0.1 mM NO_3^-, 5 mM NO_3^- or 5 mM NH_4^+) after treatment with sterile H_2O or inoculation with the fungus (Abra43).

(a)

T50	0.1 mM NO_3^-		5 mM NO_3^-		5 mM NH_4^+	
	H_2O	Abra43	H_2O	Abra43	H_2O	Abra43
**Col-0**	27 ± 0.34 (b, A)	26.5 ± 0.32 (b, AB)	26.7 ± 0.19 (b, A)	26.8 ± 0.25 (b, A)	25.6 ± 0.22 (b, B)	25.8 ± 0.20 (b, B)
**L*er***	37.4 ± 0.94 (a, A)	36.7 ± 0.83 (a)	37.1 ± 0.62 (a, AB)	37.6 ± 0.97 (a)	34.6 ± 0.47 (a, B)	35.5 ± 0.58 (a)
**Genotype (G) effect**			$<2e^{-16}$ ***			
**Nitrogen (N) effect**			$<2e^{-16}$ ***			
**Inoculation (I) effect**			0.686			
**G x N effect**			0.458			
**G x I effect**			0.686			
**N x I effect**			0.375			
**G x I x N effect**			0.922			
**Mean of G**	**Col-0**			**L*er***		
	26.4 ± 0.13 (b)			36.5 ± 0.34 (a)		
**Mean of N**	**0.1 mM NO_3^-**		**5 mM NO_3^-**		**5 mM NH_4^+**	
	31.9 ± 1.12		32.0 ± 1.14		30.4 ± 1.00	
**Mean of I**	**H_2O**			**Abra43**		
	31.4 ± 0.88			31.5 ± 0.90		

(b)

Maximum germination (%)	0.1 mM NO_3^-		5 mM NO_3^-		5 mM NH_4^+	
	H_2O	Abra43	H_2O	Abra43	H_2O	Abra43
**Col-0**	99.7 ± 0.28	99.7 ± 0.28	99.7 ± 0.26 (a)	99.7 ± 0.28 (a)	99.7 ± 0.31	100 ± 0
**L*er***	98.1 ± 1.15	98.1 ± 0.82	97.6 ± 0.65 (b)	97.3 ± 0.68 (b)	98.7 ± 0.64	98.0 ± 1.02
**Genotype (G) effect**			$<2e^{-16}$ ***			
**Nitrogen (N) effect**			0.495			
**Inoculation (I) effect**			0.458			
**G x N effect**			0.807			
**G x I effect**			0.581			
**N x I effect**			1			
**G x I x N effect**			1			
Mean of G	**Col-0**			**L*er***		
	99.8 ± 0.10 (a)			98.0 ± 0.33 (b)		
Mean of N	**0.1 mM NO_3^-**		**5 mM NO_3^-**		**5 mM NH_4^+**	
	98.9 ± 0.38		98.6 ± 0.34		99.1 ± 0.33	
Mean of I	**H_2O**			**Abra43**		
	98.9 ± 0.28			98.8 ± 0.29		
