# Supplementary material for: Nitrogen Nutrition Modulates the Response to Alternaria brassicicola Infection via Metabolic Modifications in Arabidopsis Seedlings
Source: Plants (Basel). 2024 Feb 15;13(4):534. doi: 10.3390/plants13040534 (PMC10892011; doi:10.3390/plants13040534)
Supplement: Supplementary file 1 [file plants-13-00534-s001.zip › Table S2.pdf]

Table S2 : Seedling aerial development. (a) Average values of green area, in square millimetres, for Col-0 or L*er* seedlings grown on different nutritive media (0.1 mM NO_3^-, 5 mM NO_3^- and 5 mM NH_4^+), 14 DAI with sterile H_2O. (b) Average values of green area ratio (Healthy Area Index, HAI) between seedlings treated with H_2O and seedlings inoculated with the fungus (Abra43), for Col-0 or L*er* seedlings grown on different nutritive media (0.1 mM NO_3^-, 5 mM NO_3^- and 5 mM NH_4^+), 14 DAI.

(a)

Green area (mm^2)	0.1 mM NO_3^-	5 mM NO_3^-	5 mM NH_4^+	Genotype mean
**Col-0**	27.5 ± 1.3 (a, B)	133.6 ± 22.5 (A)	27.1 ± 2.1 (a, B)	62.7 ± 11.7
**L*er***	19.7 ± 2.1 (b, B)	93.1 ± 15.5 (A)	15.3 ± 1.3 (b, B)	42.7 ± 9.0
**Nitrogen condition mean**	23.6 ± 1.4 (B)	113.3 ± 14.3 (A)	21.2 ± 1.8 (B)	52.7 ± 7.7
**Genotype (G) effect**	0.026 *			
**Nitrogen (N) effect**	$< 2e^{-16}$ ***			
**G x N effect**	0.428			

(b)

Fungus impact (HAI, %)	0.1 mM NO_3^-	5 mM NO_3^-	5 mM NH_4^+	Genotype mean
**Col-0**	81.5 ± 4.6 (b, A)	51.8 ± 9.5 (b, B)	93.5 ± 3.2 (A)	75.6 ± 4.8 (b)
**L*er***	92.8 ± 2.0 (a, A)	71.0 ± 4.8 (a, B)	93.9 ± 2.1 (A)	85.9 ± 2.3 (a)
**Nitrogen condition mean**	87.1 ± 2.4 (A)	61.4 ± 5.6 (B)	93.7 ± 1.8 (A)	80.7 ± 2.6
**Genotype (G) effect**	$<2e^{-16}$ ***			
**Nitrogen (N) effect**	$<2e^{-16}$ ***			
**G x N effect**	0.595			
