# Supplementary material for: Nitrogen Nutrition Modulates the Response to Alternaria brassicicola Infection via Metabolic Modifications in Arabidopsis Seedlings
Source: Plants (Basel). 2024 Feb 15;13(4):534. doi: 10.3390/plants13040534 (PMC10892011; doi:10.3390/plants13040534)
Supplement: Supplementary file 1 [file plants-13-00534-s001.zip › Table S3.pdf]

<u>Table S3</u> : Ammonium and inorganic ion contents in Col-0 or L*er* seedlings grown on different nutritive media (0.1 mM NO_3^-, 5 mM NO_3^- and 5 mM NH_4^+) after treatment with sterile H_2O or inoculation with the fungus (Abra43), 14 DAI.

Inorganic ion content (µmol / g DW)	0.1 mM NO_3^-				5 mM NO_3^-				5 mM NH_4^+				Mean for each ion
	H_2O		Abra43		H_2O		Abra43		H_2O		Abra43		
	Col-0	*Ler*	Col-0	*Ler*	Col-0	*Ler*	Col-0	*Ler*	Col-0	*Ler*	Col-0	*Ler*	
**Ammonium**	15.76 ± 11.27 (B)	7.23 ± 3.67 (B)	15.88 ± 6.42 (B)	12.91 ± 4.17 (B)	8.39 ± 1.73 (B)	10.39 ± 1.94 (B)	13.08 ± 2.01 (B)	12.83 ± 1.20 (B)	58.58 ± 3.99 (b, A, β)	128.36 ± 8.82 (a, A, α)	92.01 ± 13.61 (a, A, α)	37.03 ± 8.20 (b, A, β)	34.29 ± 6.61 (c')
**Chloride**	366.52 ± 79.95 (B)	263.40 ± 90.48 (B)	221.15 ± 74.46 (B)	255.99 ± 27.77	65.89 ± 8.91 (C)	42.81 ± 3.50 (C)	121.52 ± 5.59 (B)	69.97 ± 2.74	611.63 ± 79.54 (A)	714.64 ± 39.26 (A, α)	426.59 ± 99.08 (a, A)	235.27 ± 103.24 (b, β)	282.95 ± 37.93 (a')
**Nitrate**	15.21 ± 0.36 (B)	43.19 ± 24.38 (B)	53.16 ± 48.05 (B)	42.43 ± 36.10 (B)	978.49 ± 193.70 (A, α)	1036.22 ± 86.52 (A, α)	613.76 ± 51.70 (A, β)	441.35 ± 131.19 (A, β)	6.60 ± 0.93 (B)	17.35 ± 2.46 (B)	5.24 ± 0.44 (B)	12.43 ± 2.52 (B)	272.12 ± 66.54 (a')
**Phosphate**	110.47 ± 10.41	42.99 ± 13.24	175.11 ± 19.36 (a)	93.43 ± 22.48 (b)	76.70 ± 11.43	33.51 ± 2.53	100.40 ± 50.23	41.97 ± 5.26	97.78 ± 13.31	90.71 ± 4.82	98.46 ± 60.54	62.28 ± 29.05	85.32 ± 8.92 (bc')
**Sulfate**	175.75 ± 37.45 (A)	138.14 ± 51.26	186.46 ± 46.65 (A)	157.89 ± 41.30 (A)	159.88 ± 20.09 (A)	122.21 ± 3.00	188.83 ± 3.15 (A)	141.60 ± 10.98 (A)	58.93 ± 3.84 (B)	110.66 ± 23.28	52.13 ± 18.41 (B)	32.00 ± 15.81 (B)	127.04 ± 11.01 (b')

	**Col-0 mean**	***Ler* mean**
**Ammonium**	33.95 ± 7.97	34.79 ± 10.87
**Chloride**	302.22 ± 51.14	263.68 ± 57.13
**Nitrate**	278.74 ± 96.82	265.50 ± 94.09
**Phosphate**	109.82 ± 13.89 (a)	60.82 ± 8.01 (b)
**Sulfate**	137.00 ± 16.91	117.08 ± 14.19

	**0.1 mM NO_3^- mean**	**5 mM NO_3^- mean**	**5 mM NH_4^+ mean**
**Ammonium**	12.95 ± 3.19 (B)	11.17 ± 0.94 (B)	78.99 ± 10.89 (A)
**Chloride**	276.77 ± 34.89 (B)	75.05 ± 9.01 (C)	497.03 ± 65.83 (A)
**Nitrate**	38.50 ± 14.47 (B)	767.45 ± 92.51 (A)	10.41 ± 38.50 (B)
**Phosphate**	105.50 ± 15.99 (A)	63.15 ± 13.71 (B)	87.31 ± 15.29 (AB)
**Sulfate**	164.56 ± 19.76 (A)	153.13 ± 8.91 (A)	63.43 ± 11.34 (B)

	**H_2O mean**	**Abra43 mean**
**Ammonium**	38.12 ± 10.91	30.62 ± 7.56
**Chloride**	344.15 ± 64.86 (α)	221.75 ± 35.71 (β)
**Nitrate**	349.51 ± 116.82 (α)	194.73 ± 62.29 (β)
**Phosphate**	75.36 ± 7.68	95.27 ± 16.03
**Sulfate**	127.59 ± 13.47	126.49 ± 17.81

	**Nitrogen (N) effect**	**Inoculation (I) effect**	**Genotype (G) effect**	**N x I effect**	**N x G effect**	**I x G effect**	**N x I x G effect**
**Ammonium**	$1.17e^{-13}$ ***	0.215	0.857	0.014 *	0.093	$1.323e^{-4}$ ***	$7.586e^{-7}$ ***
**Chloride**	$1.087e^{-8}$ ***	$2.973e^{-3}$ **	0.308	$1.307e^{-3}$ **	0.994	0.414	0.075
**Nitrate**	$1.579e^{-13}$ ***	$1.70e^{-3}$ **	0.765	$1.036e^{-4}$ ***	0.778	0.310	0.533
**Phosphate**	0.101	0.209	$4.051e^{-3}$ **	0.186	0.388	0.533	0.976
**Sulfate**	$5.126e^{-5}$ ***	0.947	0.235	0.213	0.312	0.467	0.578
