# Supplementary material for: Nitrogen Nutrition Modulates the Response to Alternaria brassicicola Infection via Metabolic Modifications in Arabidopsis Seedlings
Source: Plants (Basel). 2024 Feb 15;13(4):534. doi: 10.3390/plants13040534 (PMC10892011; doi:10.3390/plants13040534)
Supplement: Supplementary file 1 [file plants-13-00534-s001.zip › Table S4.pdf]

<u>Table S4</u> : Primer sequences for real-time quantitative PCR amplification.

**Gene**	**Forward primer sequence**	**Reverse primer sequence**
AtActine	GGTAACATTGTGCTCAGRGGTGG	AACGACCTTAATCTTCATGCTGC
AtUBQ1	TTCCTTGATGATGCTTGCTC	TTGACAGCTCTTGGGTGAAG
AtACL5	TGGGAAAATGCAAAGTGCTG	ATAACAACTTTCTCGATCGTCGTGT
AtADC1	GTGGTGATAAGGGGAACGACA	CAACCGAAATAAGACCAATTCTCAT
AtADC2	GCGATGGACCACACAGCTT	AGAACATCCGCTGAGGACTGA
AtCYP79B2	CTCGCGAGACTTCTTCAAGG	CCATAACCAACGGTTTAGCC
AtCYP81F2	TGGCTATGCGTAAACTCGTG	CCGGTAAACTTCAAAATGGTG
AtCYP83B1	TTCATGAACGAGCACAAAGG	CATTGCAATCCCAAGATGC
AtMYB51	TCGAACTTTGACGTTGATGG	CGAAATTATCGCAGTACATTAGAGG
AtPAD3	AACCCACCATTAAGGAGC	TCATAAAATATATACGGCGTCG
AtPAO1	GCTCTAACATTCTGGTGGTGACG	CCCAAACATGTCCCTGAGAACAC
AtPAO3	ACAAACCTCACGACCTCTATG	TCAAGCACACGCATCCTG
AtPAO4	GGGAACAGTGACATTCTCGAAAC	AATTGGAACCCTGCTTCTGTCTG
AtPDF1.2	TTGCTGCTTTCGACGCA	TGTCCCACTTGGCTTCTCG
AtPR1	GGTTAGCGAGAAGGCTAACTAC	CATCCGAGTCTCACTGACTTTC
AtSPDS1	GGATCCTCGCGTGAACCTT	CGTATGATCCTTCAGCAGCATTC
AtSPDS2	AATCGGTCCAGCAAAAGAGC	GGTCCAAGCGTAGTTAACAGATCC
AtSPMS	TGCTGCTGAGTTCCTCCGTA	AGCAGGACCTACGGGATCTG
AtST5A	ATGGCTGCTCGTATTGATGG	CCGCACCAAATAACAGAAGG
AtTGG1	GTTGGTGATCGGCTTCCAGA	GAGAGTTGTGCGTGAGTCCA
AtTGG2	GGCTTCACTCACCGATACCC	AGGCGTTATGTTCCTTGCGA
